# A comparative study on anti-hyperalgesia effect of MTA and Ketoprofen in inflammatory pain

**Published:** 2009-07-06

**Authors:** Fatemeh Abbasipour, Hengameh Bakhtiar, Mehdi Vatanpour, Habib Khalilkhani, Hassan Torabzadeh, Mahyar Janahmadi

**Affiliations:** 1 *Dental Student, Neuroscience Research Centre and Department of Physiology, Medical School, Shahid Beheshti University of Medical Sciences and Endodontic Department, Dental School, Islamic Azad University of Medical Sciences, Tehran, Iran.*; 2 *Department of Endodontics, Dental School, Islamic Azad University of Medical Sciences y, Tehran, Iran.*; 3 *Department of Endodontics, Dental School, Islamic Azad University of Medical Sciences, and Member of Iranian Center for Endodontic Research, Tehran, Iran.*; 4 *Endodontist, Neuroscience Research Centre and Department of Physiology, Medical School, Shahid Beheshti University of Medical Sciences, Tehran, Iran.*; 5 *Department of Dental Material Science, Dental School, Shahid Beheshti University of Medical Sciences, Tehran, Iran.*; 6 *Department of physiology, Neuroscience Research Centre, Medical School, and Iranian Centre for Endodontic Research, Shahid Beheshti University of Medical Sciences, Tehran, Iran.*

**Keywords:** Formalin test, Inflammatory pain, Ketoprofen, Mineral trioxide aggregate, Orofacial

## Abstract

**INTRODUCTION:** Mineral trioxide aggregate (MTA) is an endodontic material with different clinical applications *e.g.* root-end filling, pulp capping and perforation repair. It has been reported to possess antimicrobial and antifungal activities. The aim of this study was to examine the effect of White MTA on formalin-induced hyperalgesia in a rat with inflammatory pain.

**MATERIALS AND METHODS:** Inflammatory pain was induced by subcutaneous (SC) injection of formalin (40 µL, 2.5%) into the rat upper lip. The nociceptive behavioral responses *i.e.* shaking of the lower jaw and face rubbing were quantified. 40 µL of eugenol (50 mg/kg), WMTA (20 mg/0.2 mL) or ketoprofen were injected solely or in combination with formalin 2.5% and the behavioral responses were compared with those observed after formalin treatment alone. One-way ANOVA, Tukey were used for analysis of data.

**RESULTS**: Formalin 2.5% provoked a biphasic nociceptive response, with an early and short lasting first tonic phase followed by a second phase. Solely SC injection of either WMTA or ketoprofen (a non steroidal anti-inflammatory drug) did not stimulate any significant nociceptive behaviour. However, injection of eugenol (a pain relieving agent) induced the early phase not the tonic phase of nociceptive response. WMTA, eugenol or ketoprofen injection 20 min before formalin injection attenuated the first phase but somehow prevented the induction of the second phase of nociceptive responses which were produced by formalin. Behavioural nociceptive responses including shaking of the lower jaw and face rubbing were significantly reduced when the subject was pretreated with either WMTA or ketoprofen (P<0.001).

**CONCLUSION:** In this study, WMTA induced pain reduction by suppression of the formalininduced nociceptive response.

## INTRODUCTION

Orofacial pain may be a result of endodontic pathology (1,2). Tissue acidification occurring after dental pulp inflammation with/without periapical lesions may result into orofacial pain (3). It can also significantly contribute to postoperative pain (4). Dental materials in direct contact with the oral tissues may also cause inflammatory reactions. Mineral trioxide aggregate (MTA) is a dental material commonly used for root-end fillings, pulp capping and perforation repairs (5-11). MTA possess adequate physical (12,13), chemical (14,15) and biological (16) properties. It has been also reported to be a non-cytotoxic (15,17-19) and non-genotoxic (20,22,23) dental material. The antibacterial (23,24) and antifungal (25-27) activities of MTA have also been documented. In orofacial formalin tests on animal models, nociception was mediated by craniofacial sensory afferent neurons; assessments of the magnitude of nociceptive sensations were elicited by long-lasting supra-threshold chemical stimulus (28,29). Trigeminal nerve innervates the orofacial regions. It also carries the pain impulses of this region *i.e.* teeth and surrounding structures (30). Subcutaneous (SC) injection of dilute formalin has been shown to activate unmyelinated polymodal nociceptors (31) and produce inflammatory pain. Tissue inflammation results in release of inflammatory mediators such as prostaglandins causing hyperalgesia (32). Ketoprofen (KP) is a nonsteroidal anti-inflammatory drug (NSAID); it has inhibitory effect on inflammatory mediators (33) and provides effective analgesia in the presence of tissue inflammation. Eugenol, a phenolic dental medicament, has been widely used as a topical sedative of pain and inflammation for pulpitis and dentine hyperalgesia (34). In the present study, the suppressive effect of WMTA against formalin-induced inflammatory pain in the orofacial region of rats was investigated and compared with the analgesic effects of ketoprofen and eugenol.

**Figure 1:**
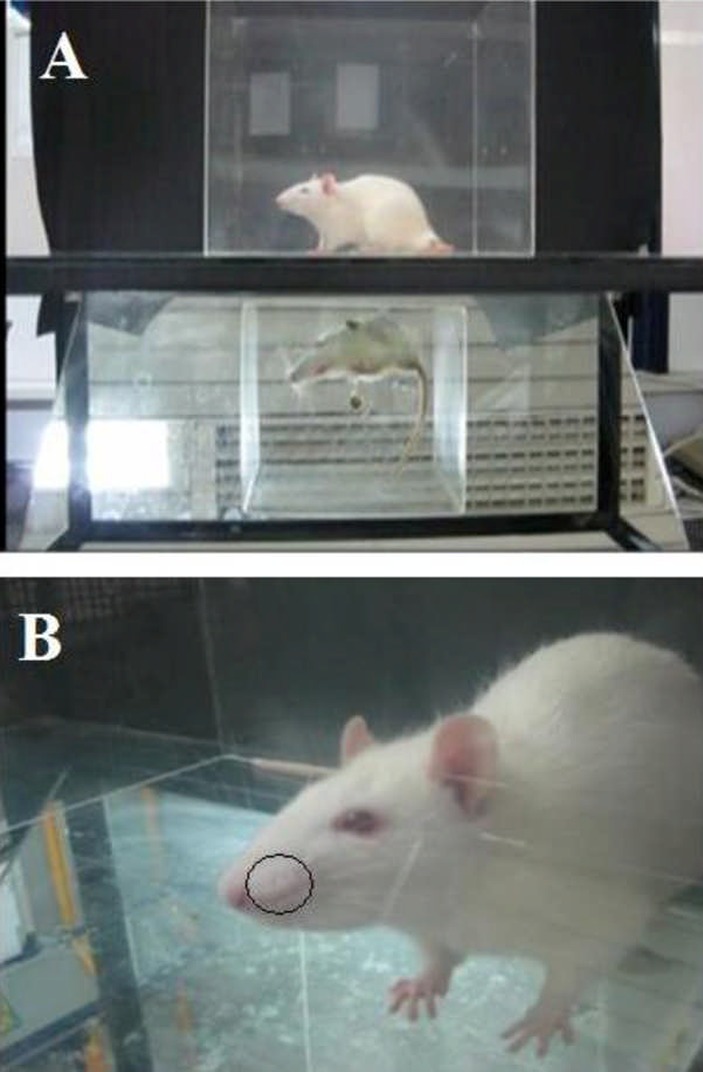
*A)* Observation box: After injection of each drug, the rat was placed in a plexiglass observation box. *B)* Injection site: Each drug was injected into the upper lip ipsilaterally, just lateral to the nose. Formalin induced inflammation at the injection site.

## MATERIALS AND METHODS

This study was approved by Ethical committee of Shahid Beheshti Medical University. Experiments were performed on male Sprague-Dawley rats (n=69, 180–200 g) housed at 23 ± 1˚C and 12-h light/dark cycles, acclimatized to the laboratory conditions for at least 72 h before use, with free access to food and water. Tests were carried out during the light phase (between 10:00 am and 5:00 pm) in a silent room. Animals were tested once and were then sacrificed at the end of experiments.

All solutions were administered by SC injection (40 µL). Drug doses were chosen based on pilot or preliminary experiments using values from the literature and our previous work (35). The 2.5% formalin was prepared by diluting the stock aqueous 37% formaldehyde solution (Sigma, UK) in 0.9% isotonic saline. MTA was prepared according to the manufacturer’s instruction.

Animals were assigned to five treatment groups: (1) formalin 2.5% (n=10); (2) injections of eugenol alone (50 mg/kg, n=10) or eugenol followed by formalin 2.5% (n=10) 20 min later; (3) WMTA (20 mg dissolved in 0.2 mL saline, n=10) alone or injection of WMTA (n=10) 20 min prior to formalin treatment; (4) ketoprofen (30 mg/kg, n=3) alone or ketoprofen followed by formalin (n=10) 20 min later and (5) saline 40µL (n=3), as a vehicle or no treatment (needle insertion only n=3).

The orofacial formalin test was performed according to Clavelou et al. (29) and Raboisson and Dallel (30). That is, before the injection, each rat was placed in a transparent plexiglass observation chamber (30×30×30 cm^3^ with a mirror placed at an angle of 45^˚^) for 30 min, in order to minimize stress-related behaviours Figure 1A. They received a SC 40 μL injection of test materials into the upper lip, just lateral to the nose Figure 1B, using a 30-gauge sterile needle. The rats were immediately returned to the transparent box for a 45-min observation. Rubbing of the injected area was regarded as the parameter of nociceptive response. Duration of nociceptive response was cumulatively recorded using a stopwatch, in consecutive 5-min intervals over a 45-min period, and was considered as an index of nociception. The nociceptive response was clearly biphasic with the first-neurogenic-phase peak occurring at approximately 5 mins after formalin injection and subsiding transiently over the next 5 mins. The second-inflammatory-phase peaked between 20-25 min. Responses that occurred during the first 5-10 min period following formalin injection were recorded as the first phase of nociception, and those occurring between 20 and 35 min as the second phase. Response scoring was performed according to Clavelou *et al.* (29). This score was based on four scales including 0 for normal behaviour *e.g.* grooming; 1, abnormal head movements; 2, abnormal continuous shaking of the lower jaw; 3, excessive rubbing of the mouth. Nociceptive scores were calculated with 5-min intervals at the end of observation, according to the following formula (T=time): Nociceptive score = [(1×T in scale 1) + (2×T in scale 2) + (3×T in scale 3)]/ 300 s (31,36).

The nociceptive behavioural responses were averaged into 5-min intervals to decrease minute by minute variability.

The results were expressed as mean ± S.E.M. Parametric tests [two tailed student *t*-test and ANOVA (analysis of variance with post hoc comparisons via Tukey’s HSD test)] and appropriate statistical software (Version 6, StatSoft, Tulsa, USA) were used. P<0.05 was considered significant.

## RESULTS

**Figure 2:**
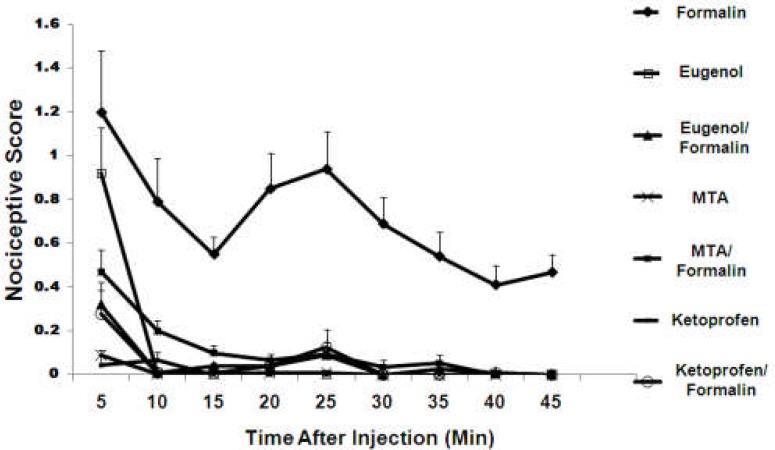
Effect of different treatments on nociceptive scores recorded in different conditions

**Figure 3:**
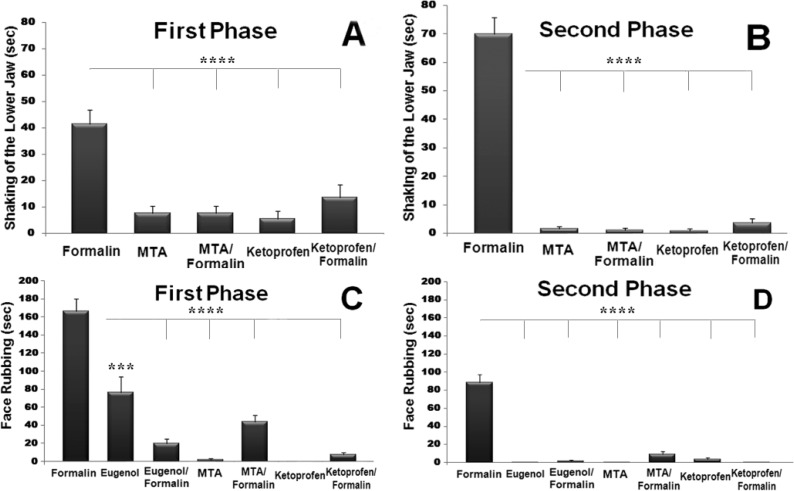
Effect of different treatments on the first (A) and the second (B) phases of shaking of the lower jaw and (C) the first phase (5-10 min post injection) and (D) the second phase (20-35 min post injection) of face rubbing nociceptive responses. Asterisks represent a significant difference as compared to formalin response

SC injection of 40 µL of formalin 2.5% elicited a typical biphasic nociceptive time course with early or short lasting phase (5-10 min) followed by a second prolonged tonic phase (20-30 min) that subsided after 45 minutes Figure 2. Rats showed shaking of the lower jaw Figures 3A and B and then sustained face rubbing episodes Figures 3C and D. Adminstration of WMTA, Eugenol and Ketoprofen before the formalin injections all statistically reduced pain.

Injection with WMTA alone did not cause nociceptive response (Figures 2 and 3). Also the injection of WMTA 20 min prior to formalin resulted in a decrease in nociceptive score (Figure 2) and significant reduction in lower jaw shaking and face rubbing in both behavior phases (P<0.001) (Figure 3). Eugenol injections either alone or 20 min before formalin treatment induced less persistent behavioral score. Injection of eugenol alone did not completely eliminate the first phase of pain behaviour but caused a significant decrease in the related face rubbing compared to formalin injection alone, while almost completely abolished the face rubbing in the second phase of nociceptive behaviour (Figures 3A and B). Ketoprofen, a NSAID, when administered 20 min before formalin injection elicited a strong inhibitory effect on the hyperalgesic reaction caused by formalin during the both phases of pain responses (Figures 2 and 3). Interestingly, no significant difference was observed between the suppressive effects of ketoprofen and WMTA (with or without formalin) both on the lower jaw shaking and face rubbing. Here, saline did not induce statistically significant nociceptive responses per se.

## DISCUSSION

The orofacial region is one of the most densely innervated areas of the body; this region is involved in nociceptive signalling (30,38). Formalin SC injection into the bilateral upper lip causes a biphasic nociceptive response which is useful for clinical pain studies (28).

The primary phases after formalin application has been attributed to a direct activation of C-fiber nociceptors; while the second and more persisted inflammatory phase is generally believed to be due to the local release of inflammatory mediators (37,39).

Information about physiological and biological aspects of root-end filling materials such as MTA is important for their clinical use. MTA has become a popular material to seal communication between the root canal system and external environment (40,41). It has also been recommended for root perforations, root end filling and apexification (42,43). MTA is commercially available in two different versions: grey-colored (GMTA) and white-colored. Both have been reported to consist of fine hydrophilic particles, but with slightly different composition (15). Both gray MTA and white MTA have been reported to produce effective antifungal (25-27) and antimicrobial (24) activities, which could be attributed to calcium hydroxide release or presumably be due to its high pH. These two factors have been reported to be responsible for the biocompatibility of MTA (8,44). It has been shown that Ca^2+^ release from MTA could be the main factor that contributed to pulp repair (45).

In the present study the exact mechanism responsible for the anti-inflammatory pain reduction of MTA has not been determined; but we can speculate that the high pH induced by MTA may be contributory. It has been shown that protons can evoke pain through acid-sensing ion channel (ASICs) activation (46), which is present in primary sensory neurons of the trigeminal nerve (47). Therefore, high pH produced by MTA may eliminate the low pH induced by inflammatory mediators.

It is suggested that tissue injury caused by direct formalin injection in the orofacial area might be as a result of the production of prostaglandin, which in turn induces inflammatory pain (32). Ketoprofen is a non-steroidal anti-inflammatory drug which effectively kills inflammatory pain in different animal models as well as humans (46,47). Our findings showed that the anti-inflammatory pain activity of white ProRoot MTA is almost comparable to ketoprofen, as an anti-inflammatory pain reducing agent. Injection of a pain relieving agent (eugenol) produced the early short-lasting pain behaviour, but not the second tonic response.

## CONCLUSION

This study suggests that white MTA does not appear to have any irritant effect on the nerve tissue and its anti-inflammatory pain reduction is comparable to ketoprofen, more effective than eugenol. MTA behaved as a palliative agent in this experimental animal model, further research is required to confirm the anti-inflammatory and analgesic properties of MTA.
